# Idiopathic calcinosis cutis of the buttocks: A case report and review of the literature

**DOI:** 10.1097/MD.0000000000031129

**Published:** 2023-04-14

**Authors:** Tian-Yu E, Xin-Jun Yang, Chen Bi, Feng Xue, Yong-Qian Cao

**Affiliations:** a Department of Plastic and Aesthetic Surgery, Shandong Provincial Hospital, Shandong First Medical University, Jinan, Shandong, China; b Department of Plastic and Aesthetic Surgery, Shandong Provincial Hospital, Cheeloo College of Medicine, Shandong University, Jinan, Shandong, China

**Keywords:** buttocks, case report, diagnosis, idiopathic calcinosis cutis, pathology, surgery

## Abstract

Calcinosis cutis is a rare skin disease, and idiopathic cases are rarely reported. It is characterized by the deposition of insoluble calcium salts in the skin, subcutaneous tissue, superficial muscles, and tendon sheaths. However, no abnormal changes were found in the bone. In this article, we introduce a case of idiopathic calcinosis cutis of the buttocks with a long course and large lesion area.

**Patient concerns:**

A 51-year-old male patient was admitted to the hospital with a chief complaint of ‘Due to the discovery of hard nodules with pruritus in the buttocks for 32 years. The patient was a male who was 51 years old. He has been in good health and reported no history of surgery, trauma, infection, metabolic disease, tumor, or other diseases. There was no family history. It is worth noting that the patient has the occupation of driving trucks, which keeps him sedentary.

**Diagnoses:**

The accurate diagnosis of calcinosis cutis was confirmed by postoperative histopathological examination with many local calcifications and multinucleated giant cells in subcutaneous tissue.

**Interventions:**

The patient underwent skin lesion excision and autologous skin grafting under general anesthesia. A medium-thickness skin graft from the left lateral thigh was transplanted into the hip operation area, and a bolus tie-over pressure dressing was applied. After the operation, the patient received anti-infection treatment and was advised to rest in the prone position to prevent extrusion of the operation area.

**Outcomes:**

The postoperative recovery was good, and there was no recurrence after 4 months of follow-up.

**Lessons:**

The incidence rate of cutaneous calcinosis is not clear. This patient had a large lesion area, long onset time, an invasion of the fat layer, and the onset site was located in the sacrococcygeal region. It is necessary to choose appropriate treatment methods.

## 1. Introduction

Calcinosis cutis is a rare and abnormal calcium deposition in the skin of all parts of the body. In 1855, Virchow reported the first case. The disease can be divided into the following five subtypes: dystrophic calcification, metastatic calcification, idiopathic calcification, iatrogenic calcification and calcium osteoporosis.^[1]^ These classifications differ in their underlying etiology, disease associations, and serum calcium or phosphate levels. The pathogenesis of calcinosis cutis is not completely clear, but scholars have put forward different theories to explain the occurrence and development of the disease. These theories provide ideas for subsequent mechanistic research and treatment. At the same time, there is no unified standard for treating calcinosis. Although many diverse treatment methods, such as drug treatment, laser therapy, and surgical treatment, this disease is still difficult to control. This paper reports a case of Idiopathic calcinosis cutis of the buttocks for a long duration and with a diffusely affected area, invading the fat layer and accompanied by pruritus. It recovers well after surgical treatment. Calcinosis is often associated with autoimmune connective tissue diseases such as scleroderma, and idiopathic calcinosis cutis is rare.

## 2. Case report

### 2.1. Case presentation

A 51-year-old male patient was admitted to the hospital with a chief complaint of ‘Due to the discovery of hard nodules with pruritus in the buttocks for 32 years. Thirty-two years ago, the patient developed a sacrococcygeal tubercle of unknown inducement, with a white in duration of the size of a grain of rice. He was treated at a local hospital and received local injections of drugs. The specific drugs used are unclear. Then, it gradually expanded into plaques, with multiple white hard nodules scattered on the plaques, accompanied by pruritus and no obvious tenderness or ulcer. In 2015, the patient underwent a pathological biopsy at the local dermatological hospital and was diagnosed with skin calcium deposition. In the past, he was healthy and had no history of trauma, infection, metabolic disease, tumor, etc. It is worth noting that the patient is a truck driver. His work requires him to sit for a long time. The patient was a male who was 51 years old. He has been in good health and reported no history of surgery, trauma, trauma, infection, metabolic disease, tumor or other diseases. There was no family history. It is worth noting that the patient has the occupation of driving trucks, which keeps him sedentary.

**Figure 1.**
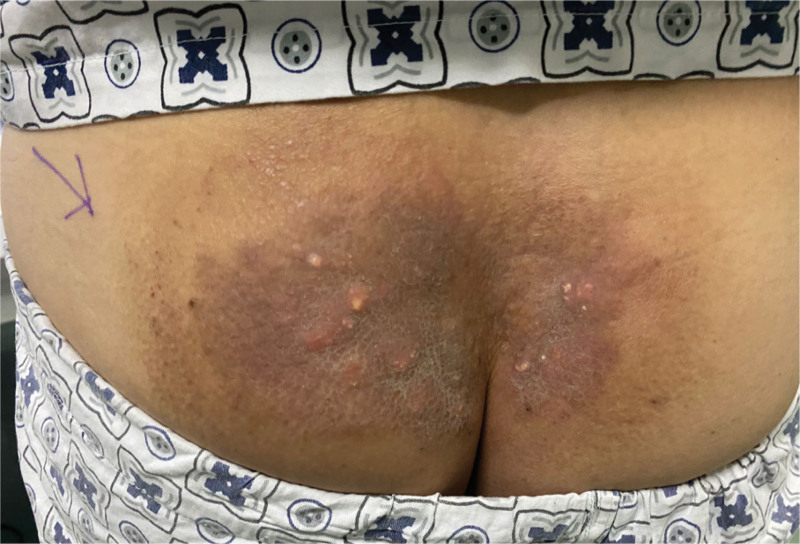
Sacrococcygeal visible 15 cm × 8.5 cm reddish brown plaque, unclear boundary, hard texture, scattered white hard nodules, adhered to the skin, without ulcer and scab.

**Figure 2.**
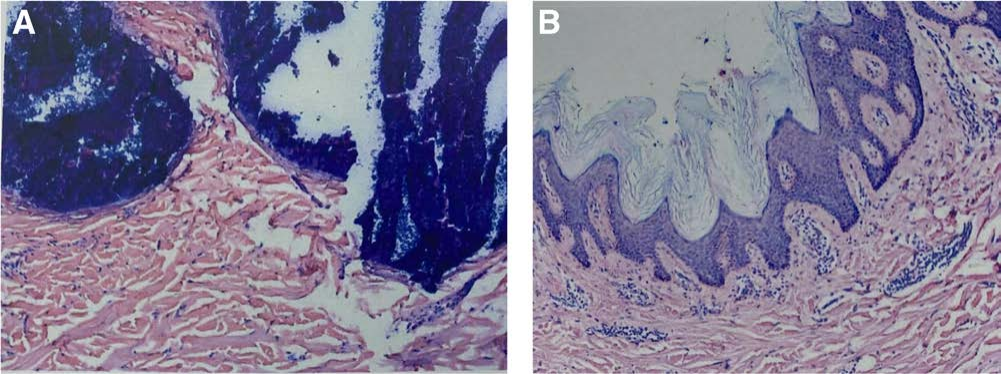
Sacrococcygeal epidermis is hyperkeratosis, spinous layer hyperplasia and hypertrophy, calcium deposition can be seen in the dermis, which is dyed dark blue by hematoxylin eosin, collagen fiber tissue hyperplasia, multiple regional calcifications can be seen in the subcutaneous tissue, with less aggregation of many nuclear giant cells. H&E staining (×400 magnification). H&E staining = hematoxylin-eosin staining.

**Figure 3.**
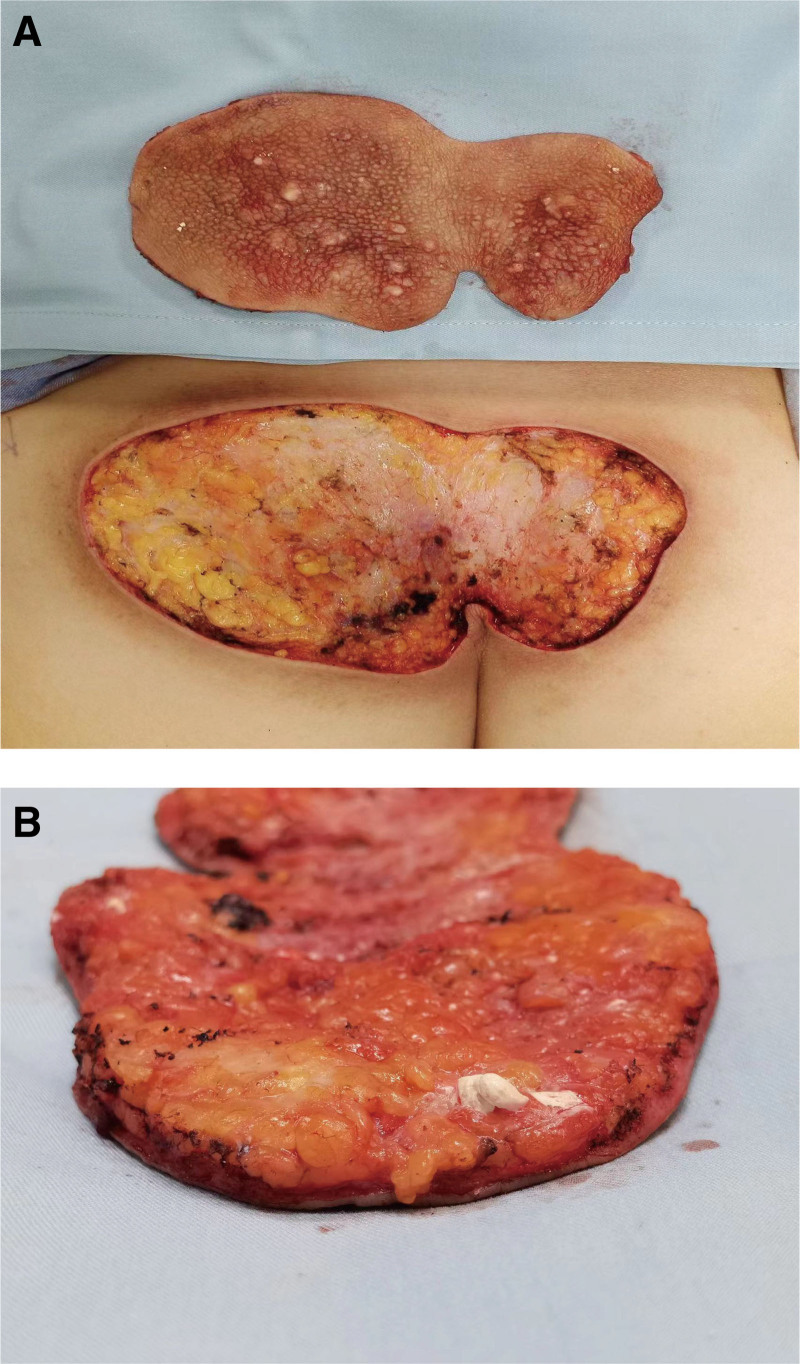
(A) Completely resected diseased tissue. (B) The lesion invades the fat layer and flows out chalk like material.

**Figure 4.**
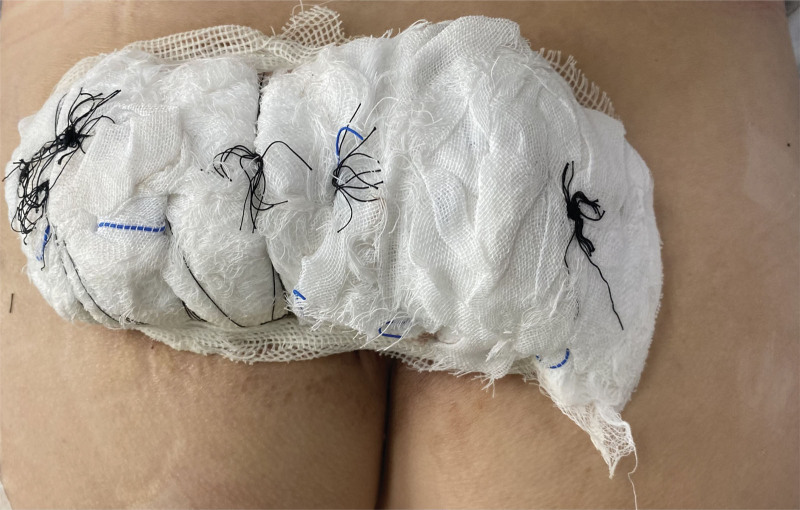
Bolus tie-over pressure dressing.

**Figure 5.**
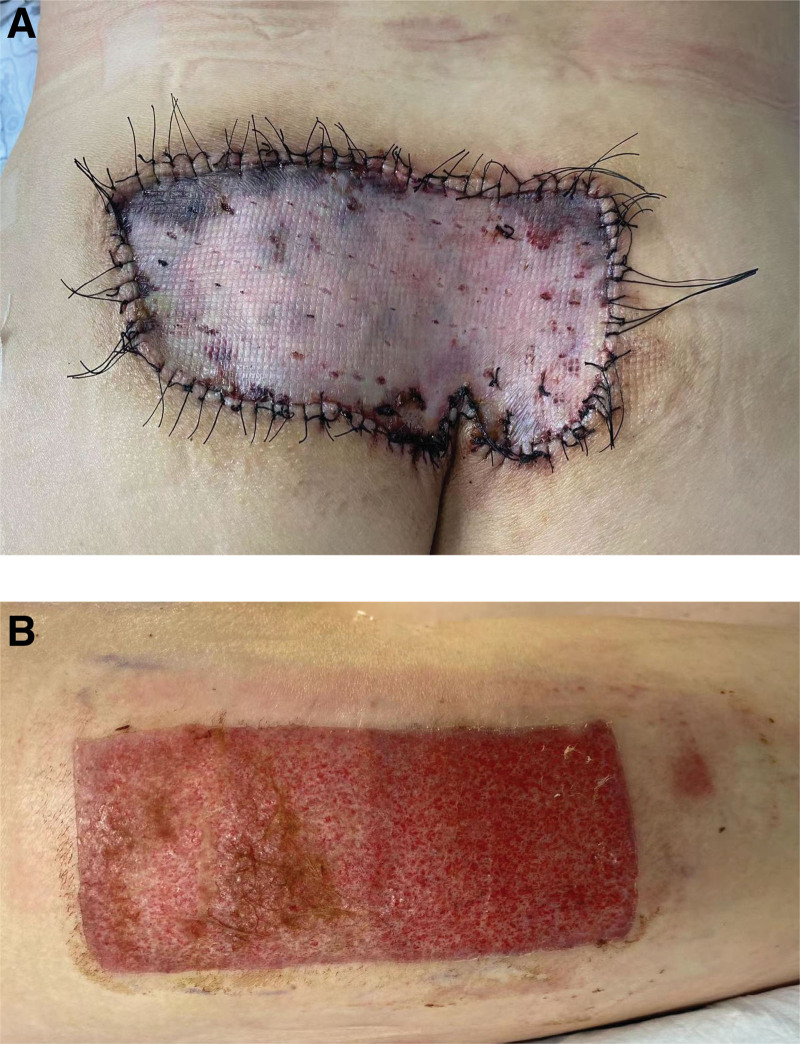
Skin graft area and donor area on the 10th day after operation.

**Figure 6.**
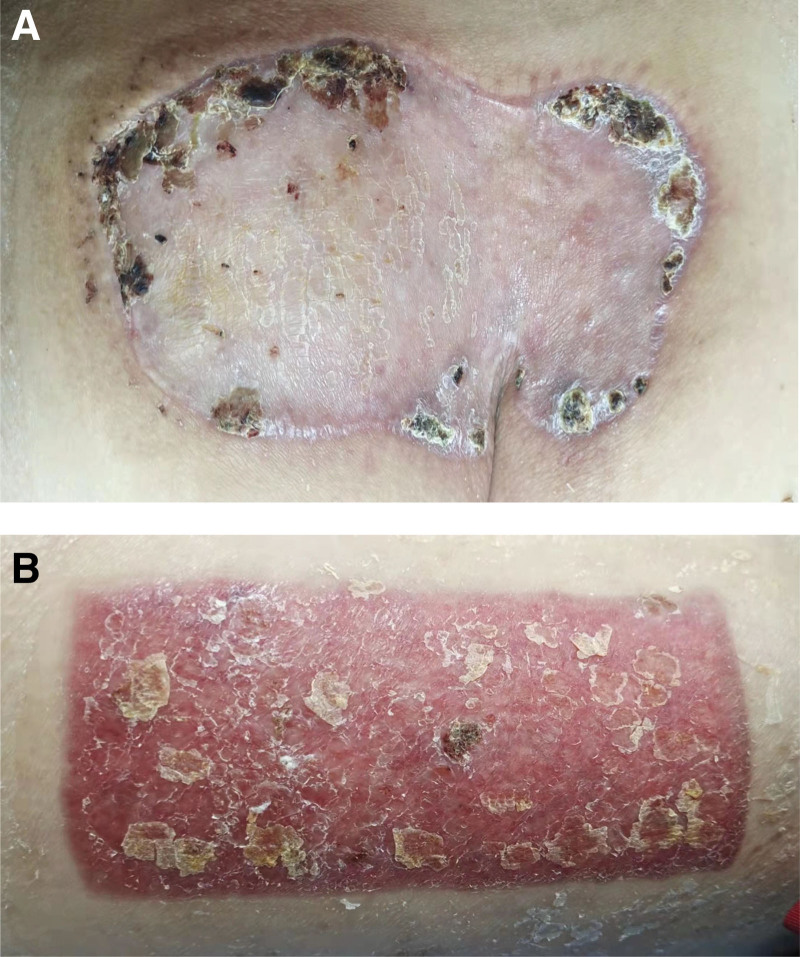
Follow up photos of one month after operation.

**Figure 7.**
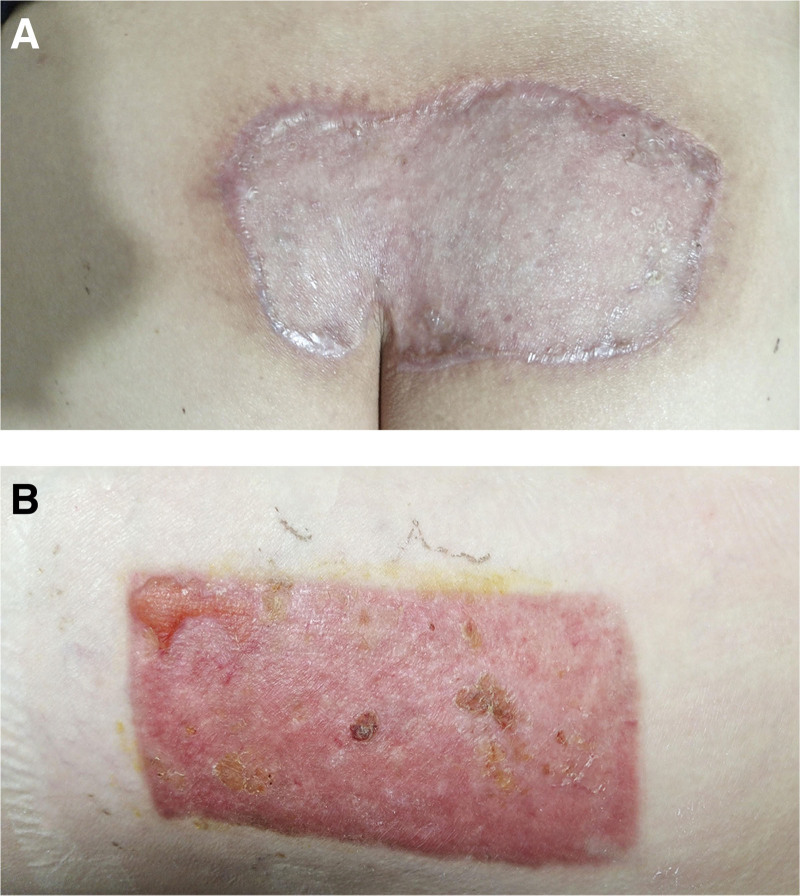
Follow up photos of three months after operation.

Physical examination: sacrococcygeal visible 15 cm × 8.5 cm reddish-brown plaque with unclear boundary and hard texture, scattered with milky white hard nodules, adhered to the subcutaneous tissue without ulceration and scab (Fig. 1). Routine blood work and biochemical labs were drawn, including liver and kidney function tests. The patient had normal thyroid function, and the parathyroid hormone levels were normal. Blood levels were as follows: calcium 2.39 mmol/L (normal value 2.2‐2.7 mmol/L), and blood phosphorus 1.11 mmol/L (0.83‐1.48 mmol/L), blood alkaline phosphatase 104 U/L (23‐140 U/L), parathyroid hormone 37.35 pg/mL (normal value 15‐665 pg/mL), Anti-ana was <1:80, and anti-double-stranded DNA was negative. The accurate diagnosis of calcinosis cutis was confirmed by postoperative histopathological examination with many local calcifications and multinucleated giant cells in subcutaneous tissue (Fig. 2). The patient underwent skin lesion excision and autologous skin grafting under general anesthesia. The nodules invaded the adipose layer and chalk-like secretions were present. The whole layer of the skin and part of the subcutaneous tissue were completely removed (Fig. 3). A medium-thickness skin graft from the left lateral thigh was transplanted into the hip operation area, and a bolus tie-over pressure dressing was applied (Fig. 4). After the operation, the patient received anti-infection treatment and was advised to rest in the prone position to prevent extrusion of the operation area (Fig. 5). After treatment, the focus of the patient was completely cleared. The postoperative recovery was good, and there was no recurrence after four months of follow-up (Figs. 6 and 7). The patient was very satisfied with the therapeutic effect.

## 3. Discussion

Calcinosis cutis is a rare skin disorder that can be secondary to various diseases, such as connective tissue disease, tumors, and trauma. It is characterized by nodules or plaques of different sizes. In the beginning stages, the skin is normal and supple. There is no pain and no adhesion damage. The mass adheres to the overlying skin, causing redness, swelling, pain, or tenderness. Finally, it will break, fester, and secrete characteristic calcareous, creamy, or purulent substances. The deposited calcium salt is mainly amorphous phosphate, a small amount of calcium carbonate and a small amount of hydroxyapatite. Combined with histopathology and laboratory examination, it is easy to diagnose. In histopathological examination, calcium deposition was stained dark blue by hematoxylin-eosin and black by von Kossa. Laboratory examination is also one of the important means of diagnosis. If some indicators are abnormal, other diseases with calcinosis cutis need to be considered. For example, hyperparathyroidism leads to calcium and phosphorus metabolism indices and connective tissue diseases such as abnormal parathyroid hormone, scleroderma, systemic lupus erythematosus and dermatomyositis. Most of them will have characteristic changes in skin and positive autoantibodies; there are definite causes of calcinosis cutis caused by intravenous calcium salt, trauma and infection. Therefore, when diagnosing this disease, we should carefully ask about the medical history, improve the relevant examination, and make a clear diagnosis according to the examination results to provide a basis for follow-up treatment.

In this case, idiopathic calcinosis cutis can be diagnosed according to the hard papules or nodules in the skin and subcutaneous tissue and the chalk-like substances discharged after rupture, combined with the patient’s medical history, histopathology and laboratory examination. The patient is a truck driver, so he must sit during work for extended periods. We speculate that long-term friction compression in the sacrococcygeal region results in pressure ischemia, which may be an important factor in the sustainable development of the patient’s condition. Compared with the small and scattered calcification in other cases, this case is characterized by a large lesion area. The course of the disease is more than 30 years, and the effect is good after large-area resection and skin grafting.

Although the pathophysiological mechanism of the disease is not precise, there are also some recognized factors, such as chronic inflammation, vascular hypoxia, trauma, and abnormal bone matrix protein. It has been reported that inflammation plays a role in calcinosis cutis.^[2]^ In juvenile dermatomyositis, the levels of serum interleukin-1, interleukin-6, interleukin-1B and tumor necrosis factor-A in patients with calcinosis are increased, and the denatured protein-bound phosphate of necrotic cells at the inflammatory site is increased, resulting in the formation of calcium deposition nests.^[3]^ There is also evidence that vascular ischemia impacts the development of calcinosis cutis. In skin biopsies of patients with systemic sclerosis with calcinosis, the expression of hypoxia-related glucose transporter molecule 1 increased,^[4]^ and the level of vascular endothelial growth factor, an effective angiogenic factor induced by hypoxia.^[5]^ The imbalance between hypoxia-induced angiogenic factors (such as vascular endothelial growth factor) and antiangiogenic factors (such as angiostatin) is common in the pathogenesis of tissue fibrosis and calcinosis cutis.^[4–6]^

In our case, the patient underwent surgery. Before treatment, the attending physician explained various treatment options, such as conservative treatment, staged resection after expander implantation, lesion resection and skin grafting. Because the patient could not bear the life impact caused by local itching symptoms and lesions, the patient chose surgical resection and skin grafting. The lesion area of the patient was large, approximately 15 cm × 8.5 cm. We discovered that the consolidation invaded the fat layer during the operation, and the calcium nodules had chalk-like secretions. The lesion area was completely removed, and the left thigh lateral medium thickness skin graft was transplanted to the operation area, packaged and sutured (Fig. 5). The operation went well. The patients were very satisfied with the operation and postoperative care. For example, in this case, the patient’s lesion was limited to one site, and the lesion range was extensive. Surgical resection is the first choice for treatment. When a patient has a small diffuse lesion, it is usually not suitable for surgical treatment. Of course, the treatment varies from person to person. Although there is no unified treatment standard, different treatment methods have been reported. Surgical treatment is the preferred strategy. Of course, drug treatment can also be selected. Since inflammation has been proven to play an important role in developing calcinosis cutis, anti-inflammatory drugs can be used to treat the disease. Colchicine, minocycline and ceftriaxone can reduce calcification and reduce symptoms.^[7,8]^ Abnormal calcium and phosphorus metabolism in vivo is also an important mechanism for the occurrence and development of skin calcium deposition disease. Therefore, regulating abnormal calcium and phosphorus levels is one of the goals of drug therapy. Diltiazem can stabilize or precipitate sediments.^[9–11]^ Aluminum hydroxide can interact with phosphorus to form aluminum phosphate and reduce the intestinal absorption of phosphorus.^[12]^ Probenecid may be associated with increased renal calcium and phosphorus excretion and decreased systemic phosphorus levels.^[13,14]^ Bisphosphonates can inhibit the transformation and remodeling of calcium and inhibit the production of proinflammatory cytokines in macrophage lines.^[15]^ This effect may play a role in inhibiting ectopic calcification. There are other drug treatments. Intravenous immunoglobulin is used clinically. It is an immunomodulator commonly used to treat autoimmune diseases.^[16]^ Sodium thiosulfate and its metabolite sodium pyrosulfite can also be used to treat calcium deposition. Warfarin can inhibit protein vitamin K-dependent γ-carboxylation to inhibit mineralization and ossification.^[17,18]^ In addition to traditional surgical treatment, laser therapy and extracorporeal shock wave lithotripsy can also be used to treat calcinosis cutis. Compared with traditional surgery, laser treatment results in less trauma and faster recovery.^[19,20]^ Small calcifications can usually be completely removed in one treatment. Isolated small calcifications can be treated with laser first.^[20]^ But common side effects are skin discoloration or scarring. Extracorporeal shock wave lithotripsy is a minimally invasive surgery that can also promote wound healing with a low risk of adverse reactions.^[21]^ This method is effective for any superficial and small-area calcinosis cutis.^[21]^ However, its limitation lies in the low accessibility to some lesions.^[21–23]^

The pathophysiological mechanism of the disease is not clear. Researchers need to compare different types of calcification to more comprehensively understand the pathogenesis of calcinosis cutis and the differences between calcification patterns. To treat calcinosis cutis, patients with etiology should actively treat the primary disease to expect the improvement of local symptoms. There is insufficient evidence to support the effectiveness of these treatments. New assessment tools and outcome measures need to be developed, and studies involving randomized controlled trials to develop evidence-based therapies for this disease. At the same time, a better understanding of the pathogenesis of calcinosis is also helpful to the choice of treatment.

## 4. Conclusion

The patient, in this case, has the following clinical characteristics: there are hard nodules and plaques on the hip skin; there was no previous history of traumatic surgery, no abnormal metabolism of calcium and phosphorus, and no evidence of other diseases. Therefore, combined with clinical manifestations and histopathological changes, it is consistent with idiopathic skin and soft tissue calcinosis diagnosis. Our patient had a large lesion area, long onset time, an invasion of the fat layer, and the onset site was located in the sacrococcygeal region. In the present case, long-term physical compression on the buttocks due to immobilization was thought to cause calcinosis cutis. The incidence rate of cutaneous calcinosis is not clear.

## Author contributions

**Supervision:** Chen Bi, Feng Xue, XinJun Yang, Yongqian Cao.

**Validation:** Chen Bi, Feng Xue, Tianyu E, XinJun Yang, Yongqian Cao.

**Visualization:** Chen Bi, Feng Xue, Tianyu E, XinJun Yang, Yongqian Cao.

**Writing – original draft:** Tianyu E.

**Writing – review & editing:** Tianyu E.
